# Attention-Guided Residual Spatiotemporal Network with Label Regularization for Fault Diagnosis with Small Samples

**DOI:** 10.3390/s25154772

**Published:** 2025-08-03

**Authors:** Yanlong Xu, Liming Zhang, Ling Chen, Tian Tan, Xiaolong Wang, Hongguang Xiao

**Affiliations:** 1 School of Nuclear Science and Technology, Naval University of Engineering, Wuhan 430033, China; 2 Chongqing Pump Industry Co., Ltd., Chongqing 400030, China

**Keywords:** fault diagnosis, attention mechanism, residual module, label smoothing regularization, convolutional neural network, small samples

## Abstract

Fault diagnosis is of great significance for the maintenance of rotating machinery. Deep learning is an intelligent diagnostic technique that is receiving increasing attention. To address the issues of industrial data with small samples and varying working conditions, a residual convolutional neural network based on the attention mechanism is put forward for the fault diagnosis of rotating machinery. The method incorporates channel attention and spatial attention simultaneously, implementing channel-wise recalibration for frequency-dependent feature adjustment and performing spatial context aggregation across receptive fields. Subsequently, a residual module is introduced to address the vanishing gradient problem of the model in deep network structures. In addition, LSTM is used to realize spatiotemporal feature fusion. Finally, label smoothing regularization (LSR) is proposed to balance the distributional disparities among labeled samples. The effectiveness of the method is evaluated by its application to the vibration signal data from the safe injection pump and the Case Western Reserve University (CWRU). The results show that the method has superb diagnostic accuracy and strong robustness.

## 1. Introduction

Rotating machinery constitutes essential components within mechanical systems. Failure events in such equipment may induce substantial economic losses [1]. These failures pose a risk of causing casualties to personnel. As a result, it is of critical importance to achieve real-time condition monitoring for rotating machinery. At the same time, it is vital to implement accurate fault identification. These capabilities can directly ensure the operational reliability of the equipment [2,3,4].

Fault diagnosis has an extremely important function in ensuring the safety and the reliability of rotating machinery. For this reason, it is of great significance for rotating machinery to have accurate and effective fault diagnosis [5,6,7]. Vibration signals contain a large amount of information. This information can reveal the safety status of the monitored system. Because of this, methods based on vibration signals are among the most commonly used tools in the reliability monitoring of rotating machinery. Nevertheless, the first two categories depend too much on the prior knowledge of experts. They also extract features in a manual way. This makes it hard to process large-scale data and learn advanced features. At the same time, when facing industrial data that is complex and changeable, it is difficult for ordinary shallow machine learning models to achieve ideal results.

In recent years, deep learning has developed rapidly. Deep learning algorithms are represented by various neural networks. These algorithms have strong feature extraction capabilities. They can conduct automatic representation learning from multiple types of data. Also, they have strong adaptability. There are some deep learning models. For example, there are stacked auto-encoders (SAEs) [8], deep belief networks (DBNs) [9], convolutional neural networks (CNNs) [10], and recurrent neural networks (RNNs) [11]. These models are widely applied in the fault diagnosis of rotating machinery. Praveenkumar et al. [12] took steps to improve classification accuracy. They did this by optimizing unsupervised algorithms. These algorithms include auto-encoders and stacked auto-encoders. They made use of the unique characteristics of acoustic emission signals and overcame the gradient disappearance problem. Zhao et al. [13] proposed a new method for fault diagnosis of rolling bearing, which uses wavelet packet decomposition (WPD) for feature extraction and chaotic sparrow search optimization algorithm (CSSOAs) to optimize the parameters of the deep belief network (DBN). This method shows the stronger feature extraction ability and excellent fault diagnosis ability of rolling bearing.

Furthermore, fault diagnosis under small-sample conditions has emerged as a significant research priority. This development reflects evolving demands in industrial applications. Chai et al. [14] proposed a Multi-scale Residual Parametric Convolutional Capsule Network (MRCCCN) that addressed small-sample feature extraction limitations through multi-segment residual convolution and dynamic routing-enhanced capsule structures. Ding et al. [15] developed Channel Attention Siamese Networks (CASNs) that resolved data scarcity limitations in critical machinery diagnostics through contrastive metric learning. Their framework enabled accurate fault identification under extreme small-sample conditions by mapping feature disparities between sample pairs and predicting unlabeled faults via distance-based classification. Gao et al. [16] introduced a Multiscale Physics-Informed Network (MPINet) that mitigated data scarcity constraints in bearing diagnostics through domain-specific physical constraints. Their framework enhanced small-sample diagnostic efficacy by encoding failure-mode-specific physical knowledge into independently trained blocks and integrating multiscale features via adaptive classification. Wen et al. [17] devised a Siamese Neural Network framework with multi-stage training that addressed data scarcity and training stagnation in motor bearing diagnostics. Zhou et al. [18] proposed a novel semi-supervised DCGAN framework that significantly enhances gear fault diagnosis with scarce labeled data by architecturally optimizing discriminator–generator balance to improve feature extraction from limited labeled samples. Liu et al. [19] proposed ICoT-GAN, a novel data augmentation framework integrating convolutional local feature extraction and transformer-based global interaction modeling, to address the challenge of global–local feature coupling under limited data. Li et al. [20] develop a label-guided contrastive learning framework with weighted pseudo-labeling (LgCL-WPL) that jointly optimized hybrid contrastive losses and classification objectives during pre-training, while enabling simultaneous utilization of labeled/unlabeled data in fine-tuning through noise-robust pseudo-labeling. Han et al. [21] introduced a pairwise sample alignment framework that enabled effective cross-domain fault diagnosis under extreme target data scarcity (1–5 samples), resolving the dual challenges of distributional discrepancy and label space mismatching through individualized domain adaptation. Their approach enhanced feature discriminability under small-sample conditions through multi-source sensor fusion, GAN, transfer learning, and other semi-supervised learning methods, validating diagnostic efficacy on industrial bearing datasets.

These studies addressed small-sample limitations through feature space refinement via domain knowledge integration, or learning strategy innovation using transfer optimization, or information utilization optimization through sensor fusion. For vibration signals, insignificant features may contain rich information, but these insignificant features are easily lost in fault diagnosis. Compared with CNN, transformers can solve this problem.

The transformer constitutes a novel neural architecture. It employs the self-attention mechanism. This mechanism was introduced by Google researchers in 2017 [22]. Self-attention operates within cost-sensitive learning frameworks. These frameworks estimate fused features through adaptive weight assignment. In order to pursue more accurate and stable diagnosis performance, some studies have preliminarily explored and applied the method of combining the attention mechanism in transformers with CNNs. Wang et al. [23] proposed a lightweight CNN–transformer named SEFormer for rotating machinery fault diagnosis. This study provides a feasible strategy for developing a lightweight rotating machinery fault diagnosis framework aimed at economical deployment. Xu et al. [24] developed a new channel attention mechanism based on squeeze and excitation modules to focus on key features while reducing the computational complexity of the network. Wang et al. [25] put forward an ECA-CNN framework with multi-sensor fusion that addressed inadequate feature representation in rotating machinery diagnostics. Their approach enhanced channel-wise feature discriminability through adaptive attention weighting and multi-source data integration, achieving efficient fault identification under noisy conditions. However, these methods do not capture and assign higher weights to important long-term dependent information, nor do they have the capability to deeply mine models.

To address these issues, this paper maps the time-domain information of vibration signals onto two-dimensional images, ensuring the generalization ability and anti-interference capability of the diagnosis model, while eliminating the influence of experts’ prior knowledge on the images. LSTM is used to model the dynamic evolution of time series signals and capture long-distance causal dependence. Additionally, label smoothing regularization (LSR) is introduced to balance the distributional differences between label samples. The method is tested on a CWRU dataset and safe injection pump fault dataset. Experimental results show that the method can accurately identify two types of faults. In particular, when the safe injection pump fault dataset has few data samples, the model shows outstanding performance. The main contributions of this paper are as follows:

1. A convolutional neural network model based on an attentional mechanism and deep residual is proposed. The effects of optimizers are discussed. The method has high test accuracy. A large number of safe injection pump fault simulation experiments are carried out. The effectiveness of the method is verified by the fault data collected from safe injection pumps.
2. The sensitivities of the attention mechanism and LSTM to the ratio of training samples are discussed, where the attention mechanism can capture channel and spatial information of the vibration signal. Then, LSTM can extract the temporal features of vibration signals. In addition, visualization techniques are also used to understand blocks in AR-CLSTM.
3. Two case studies were performed to validate the proposed diagnostic framework. Experimental outcomes demonstrated that AR-CLSTM exceeded six benchmark methods. This performance advantage was particularly notable with small samples.

The remaining structure of this work is organized as follows. Section 2 presents fundamental theoretical models for fault diagnosis. Section 3 details the AR-CLSTM framework. This section also introduces advanced regularization training strategies. Section 4 validates the applicability of AR-CLSTM through two case studies. Section 5 provides concluding remarks and future research directions.

## 2. Theoretical Background

### 2.1. Convolutional Neural Networks

**Figure 1:**
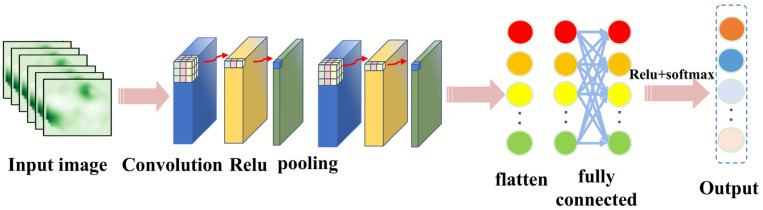
Schematic diagram of CNN structure.

CNN is a multi-stage neural network consisting of several filtering stages and a classification stage. It is inspired by the structure of the vision system, developed by LeCun and collaborators in 1990 for image processing [26], and is still widely used in computer vision application. A general CNN architecture is shown in Figure 1, which mainly consists of an input layer, convolutional layer, pooling layer (downsampling), fully connected layer, and output layer.

The convolutional layer serves as the core component within convolutional neural networks (CNNs) for performing feature extraction. This layer comprises multiple learnable convolution kernels. Each element constituting a kernel corresponds to a distinct weight coefficient; additionally, each kernel is associated with a bias term. The extracted features are subsequently propagated to the next layer of the network for processing. The size of the localized input region involved in each convolution operation is determined solely by the dimensions of the convolution kernel itself. The mathematical expression for the convolution operation is as follows:

(1) $$y_{j}^{l}=\sum_{i=0}^{k}\;x_{i}^{l-1}\times w_{j}^{l}+b_{j}^{l}$$

where $x_{i}^{l-1}$ represents the *i*th feature input of the *I*th layer, $w_{j}^{l}$ represents the *j*th weight coefficient of the *I*th layer, $b_{j}^{l}$ represents the *j*th bias of the *I*th layer, and $y_{j}^{l}$ represents the *j*th output feature of the *I*th layer.

The convolutional layer is typically succeeded by a pooling layer. This layer executes feature selection and information filtering on its input features. It reduces the number of feature parameters. This reduction eliminates redundant information. Commonly employed pooling methods include maximum pooling and average pooling. Maximum pooling sees particularly widespread application. The computational procedure for the maximum pooling layer is

(2) $$P_{m}^{l}=max\left(x_{m}^{l}\right)$$

where $P_{m}^{l}$ is the output of the *m*th area of the *I*th layer; $x_{m}^{l}$ is the *m*th area of the pooled *I*th layer.

In order to reduce the offset of interval covariance, a batch normalization layer is introduced by reducing the calculation load to improve the learning speed. It is usually added after the convolution layer or before the activation layer. Normalized transformations can be described as

(3) $${\hat{y}}^{l\left(i,j\right)}=\frac{y^{l\left(i,j\right)}-\mu_{B}}{\sqrt{\left(\sigma_{B}^{2}+\varepsilon \right)}}$$

(4) $$z^{l\left(i,j\right)}=\gamma^{l\left(i\right)}{\hat{y}}^{l\left(i,j\right)}+\beta^{l\left(i\right)}v$$

Among them, $z^{l(i,j)}$ is the output of a neuron response, $\mu_{B}=E\left[y^{l\left(i,j\right)}\right],\;\sigma_{B}^{2}=Var[y^{l(i,j)}]$, ε is a small constant added to the numerical stability, and $\gamma^{l(i)}$ and $\beta^{l(i)}$ are the scale and displacement parameters that need to be learned, respectively.

Generally, the activation function is used to process the features extracted by the convolutional layer to enhance the feature expression ability of CNN. The activation function improves the nonlinear mapping capability of the model by mapping originally linearly inseparable multidimensional features to another space. Commonly used activation functions include Sigmoid, Tanh, ReLU, etc.

### 2.2. Long Short-Term Memory (LSTM)

**Figure 2:**
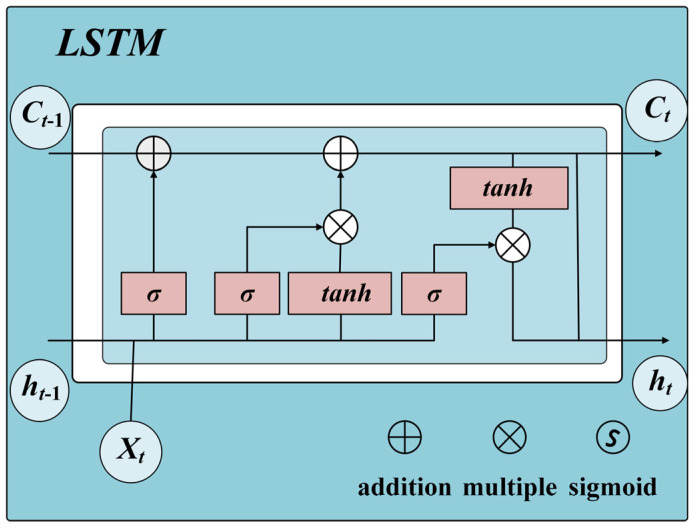
The structure of LSTM.

Recurrent neural networks (RNNs) are widely used in sequence learning, but the problem of vanishing gradients in training backpropagation steps hinders their performance. To avoid this obstacle and capture the long-term dependence of data characteristics, Hochreiter et al. [27] have improved RNN into a new architecture called long short-term memory (LSTM). It shows more efficient classification and regression performance than RNN on sound and natural language processing datasets. The structure of LSTM is shown in Figure 2.

The LSTM network consists of one or more LSTM units used to capture long-term dependencies in time series data.

(5) $$i_{t}=\sigma \left(W_{i}*\left[h_{t-1},x_{t}\right]+b_{i}\right)$$

(6) $$f_{t}=\sigma \left(W_{f}*\left[h_{t-1},x_{t}\right]+b_{f}\right)$$

(7) $$o_{t}=\sigma \left(W_{o}*\left[h_{t-1},x_{t}\right]+b_{o}\right)$$

(8) $$C_{t}=f_{t}*C_{t-1}+i_{t}*tan\;h\left(W_{c}*\left[h_{t-1},x_{t}\right]+b_{c}\right)$$

(9) $$h_{t}=o_{t}*tan\;h\left(C_{t}\right)$$

In these formulas, $i_{t}$, $f_{t}$, and $o_{t}$ are the activation values of the input gate, forgetting gate, and output gate at time step *t*; $C_{t-1}$, $C_{t}$, and $h_{t}$ are the cell state at the previous *t* − 1, the cell state at current *t*, and the hidden state at current *t*, respectively; σ is the activation function; $W_{i}$, $W_{f}$, $W_{o}$, and $W_{c}$ are the weight matrices of the input gate, forgetting gate, output gate, and cell states, respectively; $\left[h_{t-1},x_{t}\right]$ connects the hidden state of the previous moment $h_{t-1}$ and the input $x_{t}$ of the current moment into a vector; $b_{i}$, $b_{f}$, $b_{o}$, and $b_{c}$ represent biased vectors of the input gate, forgetting gate, output gate, and cell states, respectively; tanh is a hyperbolic tangential activation function; and $tan\;h\left(C_{t}\right)$ applies a hyperbolic tangent activation function to the current cell state.

### 2.3. Residual Neural Network

Theoretical analysis shows that making networks deeper usually improves their ability to represent features. However, experimental results demonstrate a degradation effect. When network depth passes certain critical points, generalization performance declines. This decline occurs even though architectural complexity increases. To solve this key optimization challenge, He et al. [28] proposed the residual learning concept. They developed the important ResNet architecture using this concept.

This framework uses residual blocks as basic building units. Every block contains several operations in sequence. These typically include convolutional layers, batch normalization, and rectified linear unit activation functions. Crucially, identity skip connections integrate with these operations.

**Figure 3:**
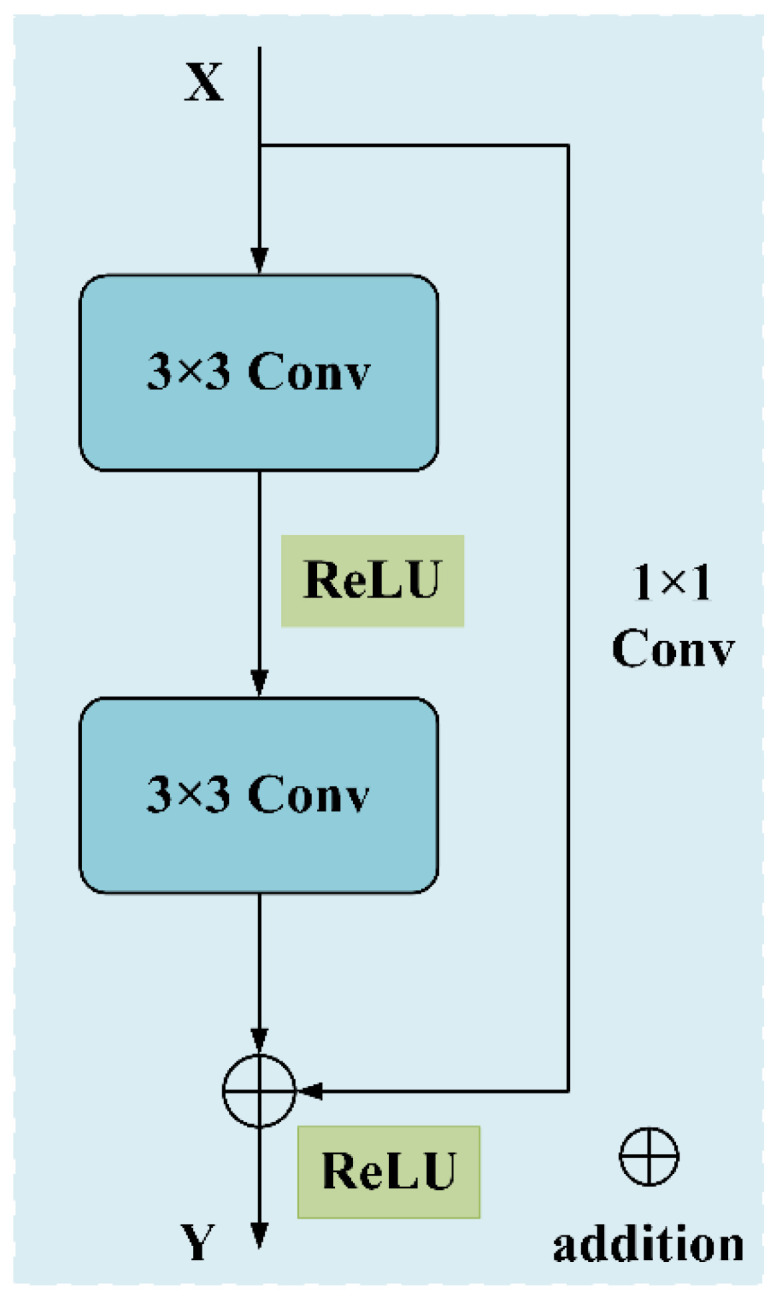
The structure of residual block.

For a given input $x_{l}$, the output of the *l*th residual block can be expressed as

(10) $$x_{l+1}=f\left(h\left(x_{l}\right)+F\left(x_{l},W_{l}\right)\right)$$

where $h\left(x_{l}\right)$ is the shortcut connection; function F(·) is the residual block mapping, which represents the learned residual; $W_{l}$ is the network parameter; and f(·) is the ReLU activation function. In this paper, the designed residual neural network includes one residual block, and the structure of the network is shown in Figure 3.

## 3. Proposed Method

**Figure 4:**
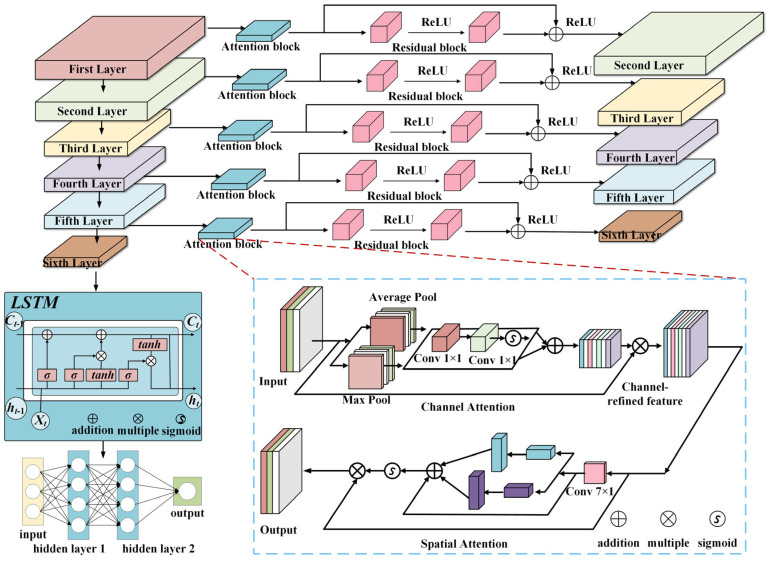
The diagram of the proposed AR-CLSTM.

Figure 4 shows the pipeline of our proposed AR-CLSTM model. The AR-CLSTM architecture integrates convolutional networks, attention mechanisms, residual connections, and LSTM units. This integration enables temporal–spatial feature extraction. Six convolutional stages form the structural foundation. Each stage contains a standard convolutional operation. Batch normalization follows this convolution. The LeakyReLU activation functions apply nonlinear transformations. Channel attention modules operate after activation. These modules compute adaptive channel-wise weighting. Spatial attention mechanisms then execute feature recalibration. Both attention types cooperate for feature optimization. Residual blocks connect the initial five stages. These residual connections mitigate gradient dissipation. Channel attention prioritizes significant frequency components. Weight assignment occurs through learned importance measures. Spatial attention identifies critical feature regions. Adaptive optimization results from their combined operation. LSTM outputs feed into fully connected layers. Classification occurs through these subsequent layers. ReLU activations introduce nonlinear decision boundaries. Dropout regularization prevents model overfitting.

### 3.1. CLSTM

**Figure 5:**
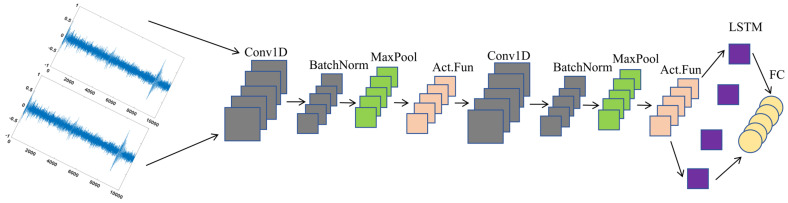
Schematic diagram of CLSTM structure.

By combining the convolutional layer explained in Section 2.2 with the LSTM architecture, we obtain a convolutional LSTM (CLSTM). The convolutional layer enables the architecture to extract different types of waveform input arrays, and its abstract output is immediately processed by the LSTM layer to analyze its inherent sequential and periodic behavior. Shi et al. [29] argue that CLSTM can better capture spatiotemporal correlations than other architectures such as CNN or simple LSTM. Figure 5 draws a diagram of the CLSTM architecture.

### 3.2. Attention Mechanism

**Figure 6:**
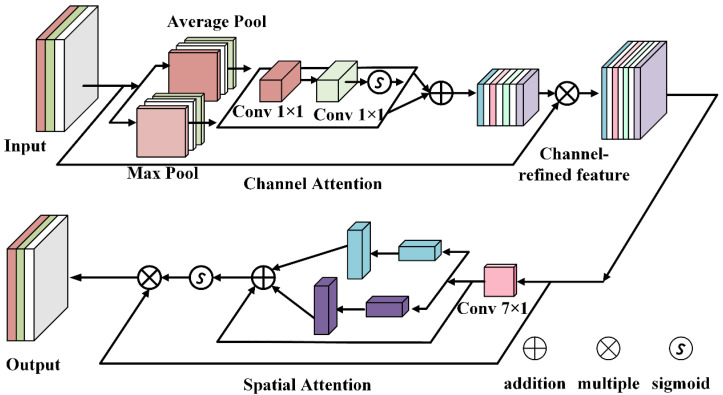
The structure of attention mechanism.

In order to reduce irrelevant background information and enhance the attention to defect features, a channel attention submodule and spatial attention submodule are introduced into the backbone network to improve its feature extraction ability. The structure of the channel attention mechanism and spatial attention mechanism is shown in Figure 6.

For the input feature graph $\;F_{1}\in R^{C\times H\times W}$, firstly, the dimension transformation is performed, and the input MLP is processed. Finally, the feature graph is transformed into the initial dimension again through the feature graph output by the MLP, and the output channel attention feature vector ($F_{1}$) is obtained by using an s-type operation, which is defined as

(11) $$M_{c}\left(F_{1}\right)=\sigma \left(permute\left(MLP\left(permute\left(F_{1}\right)\right)\right)\right)$$

where *σ* is an s-type function. Multiplying the channel attention feature vector with the input feature element yields an intermediate state $F_{2}$, which is used as the input feature of the spatial attention submodule and is defined as

(12) $$F_{2}=M_{c}\left(F_{1}\right)⊗F_{1}$$

To focus on spatial information, the spatial attention submodule then performs spatial information fusion using two convolutional layers. The number of channels is first reduced by convolution kernel 3 × 3 (from C to C/r, r = 16, representing a reduction in the number of channels), the number of channels is increased by convolution operations, and convolution kernel 3 × 3 keeps the number of channels the same; finally, the spatial attention feature $M_{s}\left(F_{2}\right)$ output can be defined as follows:

(13) $$M_{s}\left(F_{2}\right)=\sigma \left(f^{3\times 3}\left(f^{3\times 3}\left(F_{2}\right)\right)\right)$$

You can multiply the spatial attention feature vector by the input feature elements of the spatial attention submodule to obtain the output feature of the GAM attention module, and its formula is expressed as

(14) $$F_{3}=M_{s}\left(F_{2}\right)⊗F_{2}$$

where $M_{c}$ and $M_{s}$ are channel graphs and spatial attention feature graphs, respectively, and ⊗ denotes element-based multiplication.

### 3.3. Label Smoothing Regularization

Cross-entropy loss (CE, $l_{0}$) tends to focus on one direction, resulting in poor regulation ability. Therefore, adding the smoothing coefficient *ε* to increase the correct diagnosis and reduce the wrong diagnosis, which helps to combat the overconfidence of the model and improve learning ability. LSR (l) can not only upgrade generalization but can also calibrate the model. It is mainly used in the field of image recognition but is rarely used in research on fault diagnosis.

Assume that *p*(*k*) is the predicted distribution and $q\left(k\right)$ is the real distribution. The real distribution after the label is smoothed is $q^{\prime }\left(k\right)$, the coefficient is ε, the category is K, and the label distribution is set to uniform distribution *μ*(*k*) = 1/*K*. Then, the relationship between $l_{0}$ and l can be deduced, as shown in the following formula:

(15) $$l=-\sum_{k=1}^{K}\log\left(p\left(k\right)\right)q^{\prime }\left(k\right)\;=-\sum_{k=1}^{K}\log\left(p\left(k\right)\right)\left[\left(1-\varepsilon \right)q\left(k\right)+\frac{\varepsilon }{K}\right]\;=\left(1-\varepsilon \right)\left[-\sum_{k=1}^{K}\log\left(p\left(k\right)\right)q\left(k\right)\right]+\varepsilon \left[\frac{-\sum_{k=1}^{K}\log\left(p\left(k\right)\right)}{K}\right]\;=\left(1-\varepsilon \right)l_{0}+\varepsilon \left[\frac{-\sum_{k=1}^{K}\log\left(p\left(k\right)\right)}{K}\right]$$

Overfitting is mitigated by learning smooth labels rather than real labels, so we believe LSR has potential advantages in handling small samples in troubleshooting.

## 4. Results and Discussion

The proportion of each training sample (α%) was used as the evaluation criteria. We believe that if α < 0.5, it can be considered a small sample [30,31]. α ≤ 0.3 is used to simulate the scenario of extreme data scarcity (e.g., safety injection pump failure), and α > 0.3 is used to verify the generalization ability of the model from scarce to sufficient data. First, the advantages of the new regularization training method are verified. Then, when α = 0.1~0.5, the small-sample learning ability of different models is verified and performance evaluation is performed under different working conditions. Finally, parameter sharing for small-sample transfer learning applied to the new dataset will be discussed, and visual interpretation of AR-CLSTM will also be discussed. All experiments were performed under the same random conditions, and the experimental settings were shown in Table 1. All datasets underwent stratified partitioning: 70% for training and 30% for testing. Final evaluation used a completely independent test set processed with non-augmenting transformations.

The experiment is implemented in PyTorch 2.1.0, Python 3.8.7, running on AMD Ryzen 7 7840H CPU @ 3.8 GHz (16G RAM). The mini-batch size is set to eight in this study. In addition, the label-smoothing training strategy is utilized to supervise the training of the AR-CLSTM model. The structure and main hyperparameters of the AR-CLSTM model are as shown in Table 2.

### 4.1. Methods of Model Evaluation and Metrics

The AR–CLSTM framework, which is introduced in detail in Section 3, is now validated through two industrial case studies. For the Case Western Reserve University (CWRU) bearing dataset, the channel attention mechanism prioritizes the fault-related frequency bands in the vibration spectrum, while the spatial attention mechanism locates the transient impulses in the two-dimension time–frequency representation. The residual block alleviates gradient dissipation during the deep feature extraction process, and LSTM captures the temporal dependencies in the motor speed variations. For the safety injection pump (Case 2), label smoothing regularization explicitly addresses the data scarcity problem by reallocating label confidences among similar fault categories. Diagnostic performance can be represented by a confusion matrix, where it has two valuable metrics. In the case of multiple classes, this is the average of the F1 scores for each class, weighted depending on the average parameters, where sensitivity (recall) and precision are key performance indicators, defined as follows, which can be obtained directly from the confusion matrix, as shown in Table 3.

(16) $$precision=\frac{TP}{TP+FP},sensitivity=\frac{TP}{TP+FN}$$

(17) $$F_{\beta }=\frac{\left(1+\beta^{2}\right)\left(precision+sensitivity\right)}{\beta^{2}\times precision+sensitivity}\left(\beta =1\right)$$

Among them, standard metrics derived from the confusion matrix include precision (*TP*/(*TP* + *FP*)), recall (*TP*/(*TP* + *FN*)), and F1 score.

To better visually present the correct and incorrect prediction results, we used a normalized confusion matrix to evaluate the performance of the model. The elements in each cell are defined as shown in Table 4.

### 4.2. CWRU Database

#### 4.2.1. Description and Distinction of Data

**Figure 7:**
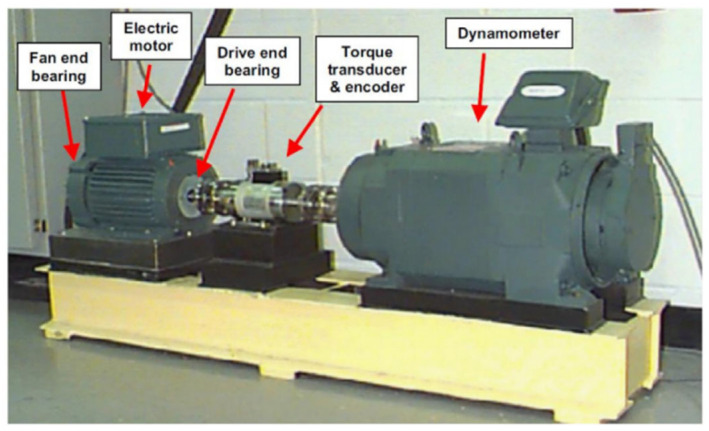
Bearing fault diagnosis model test bench.

The public dataset from CWRU is the bearing timing signal data collected by its experimental equipment, as shown in Figure 7. Due to human limitations, the motor shaft supported by the bearing does not rotate at a single approximate speed, and its speed changes with the change in loads; see Table 5 for details.

This paper selects 12 kHz for experiments, as shown in Table 6. For the drive end bearing of the SKF6205-type model (Case Western Reserve University, Cleveland, OH, USA), it is manually implanted into a single point of failure by electrical discharge machining. There are three types of fault locations: in-vehicle fault (IRF), rolling element fault (BF), and out-vehicle fault at 6 o’clock (ORF). Each fault location contains four fault dimensions: 0.007 inches, 0.014 inches, 0.021 inches, and 0.028 inches. Some data are not available, so for the sake of experiment integrity, we do not take all data with a fault size of 0.028. Therefore, in the experimental data, the bearing states under a certain load can be divided into nine fault states and one normal state, corresponding to ten domain vibration signals. Since the original data are long time series signals collected continuously, in order not to miss the fault information, we use the sliding window segmentation method to divide the entire signal into several short samples. Each fault operating condition contains 212 samples, and each sample consists of 512 non-overlapping data points. The normal state operating condition contains 840 normal samples. The entire dataset contains a total of 212 × 9 + 840 = 2748 samples.

#### 4.2.2. Discussion of Batch_Sizes

A larger batch number can shorten the training time for each iteration, but it may also reduce the generalization ability, so a balance should be achieved between the two. Therefore, under the fault dataset A when the dataset is CWRU, the batch_size is selected as 64, α = 0.4, and only the batch_size has changed. The results are shown in Table 7.

The training difficulties of different batches are not consistent, resulting in different training times. Obviously, it can achieve similar performance (99.93%, 100%) using a batch_size of 16 or 64, but the latter takes less time (130.76886 s), so the batch_size is set to 64.

#### 4.2.3. Discussion of Optimizers

Different optimizers will affect the accuracy and training time of each iteration. For a certain neural network, they are used to optimize the objective function, and the parameters are constantly updated until the optimal solution is achieved. The closer the solution is to global optimality, the better the neural network is generalized. In this paper, when the batch_size is 64, α = 0.4, and the number of iterations is 200, the results are shown in Table 8. Except for ASGD, the accuracy of the other test sets is high, while the test set accuracy of Adam, Adamp, and Adamax optimizers all reach 100%, but the Adam time is the shortest (130.7688 s).

**Figure 8:**
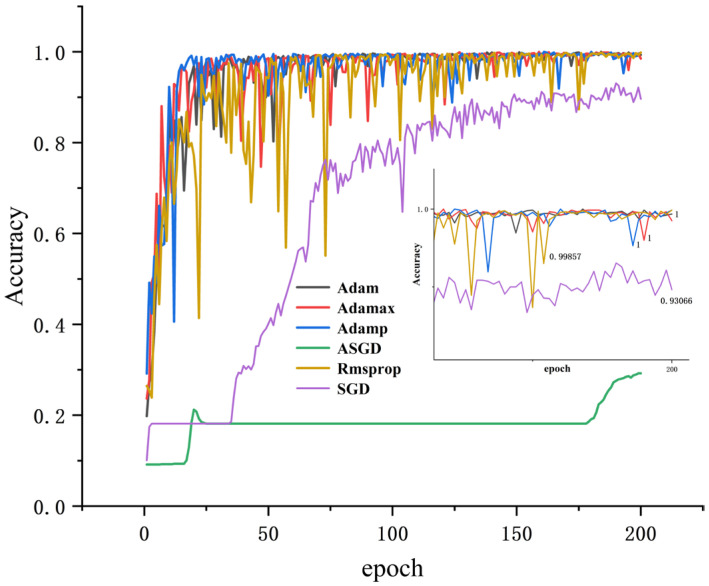
Accuracy of different optimizers.

**Figure 9:**
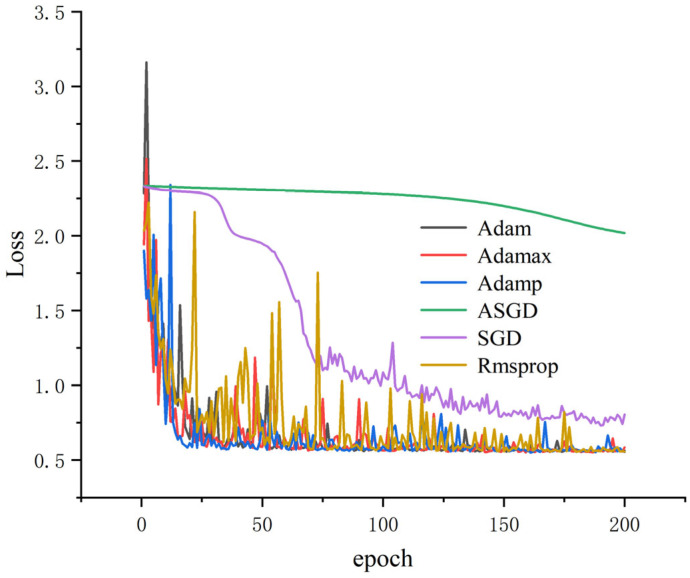
Error rates for different optimizers.

All results were obtained using dataset A, with α = 0.4, and the training loss and accuracy were obtained. As shown in Figure 8 and Figure 9, it can be seen that the accuracy of several optimization algorithms except SGD and ASGD has reached more than 99%. Rmsprop has the largest oscillation amplitude; Adamp and Adamax also have a larger amplitude, while the accuracy and loss rates of Adam tend to stabilize under shorter iterations. It can be seen that Adam performs well, so Adam is selected as the optimizer of this model.

#### 4.2.4. Comparison of Ablation Experiments

The ablation experiment of AR-CLSTM (M4) was carried out on four datasets: A, B, C, and D. The comparison models were R-CLSTM (without attention mechanism M1), A-CLSTM (without RESNET, M2), and CLSTM (without attention and RESNET, M3) with F1 scores as the indicator. A→A represents the transition from the training set to test set. The X-axis represents the ratio of training (α). At the same time, the running time of different loads under different models and different α is recorded as shown in Table 9.

**Figure 10:**
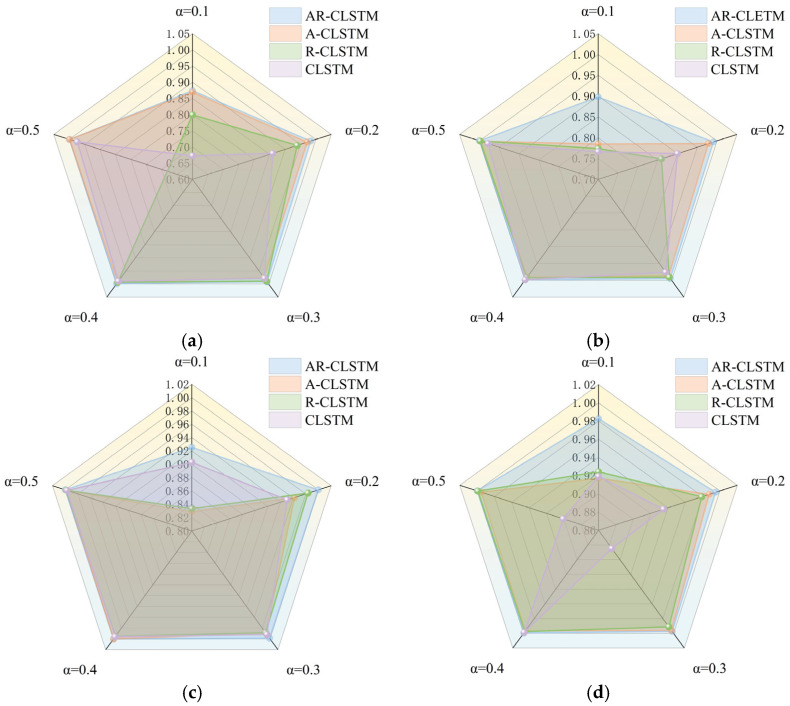
F1 scores under different loads. (**a**) A→A; (**b**) B→B; (**c**) C→C; (**d**) D→D.

As can be seen from Figure 10 and Table 9, as the model increases, the F1 scores also increase. Meanwhile, as the complexity of the model increases, the computational cost of the model also grows, and the time spent on training becomes longer. In addition, as the complexity of the model increases, the computational cost of the model also grows, and the time spent on training becomes longer. The residual module solves the degradation problem of deep networks. Under dataset A, when α = 0.1, M1 = 0.7992 and M3 = 0.6729. The F1 score of M1 is 12.63% higher than that of M3. Meanwhile, under different working conditions, the performance of M1 in the case of small samples is generally better than that of M3. From M2 and M3 in Figure 10b, when α = 0.2, M3 = 0.9006 and M2 = 0.9784; this shows that the attention mechanism has a good generalization of small samples because it can reduce the computational burden of processing high-dimensional input data, reduce the data dimension, and find significant useful information related to the current output in the input data. Combining the two, M5 = 0.9911. In general, both are beneficial to the performance of the model for small-sample cases. Furthermore, there is a trend of running time increasing with the increase in α, where the advanced model requires more time. Overall, AR-CLSTM has the highest diagnostic efficiency.

### 4.3. Fault Diagnosis of Safe Injection Pump Dataset

#### 4.3.1. Database Introduction

In this case study, the proposed method is used to diagnose faults in safe injection pumps. The safe injection pump model is CDWL25-0.4 (Chongqing Pump Industry Co., Ltd., Chongqing, China), with a rated power of 30 kW, and the rated speed of the drive motor is 1460 r/min. The INV3065N2 multi-function dynamic signal testing system and piezoelectric accelerometer INV982X were used for vibration signal acquisition, and the sampling frequency was 10 kHz in the experiment. Signal collection was completed at Chongqing Water Pump Factory [32].

**Figure 11:**
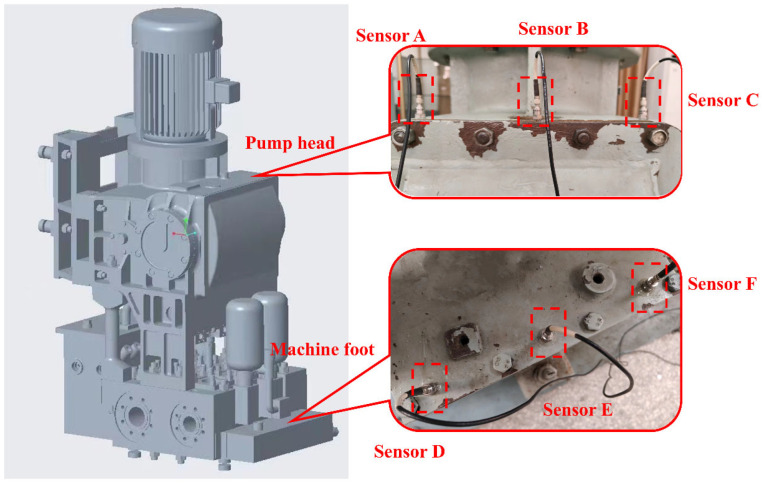
Experimental setup of safe injection pump.

As shown in Figure 11, the diagnostic object is a vertical safe injection pump, whose drive mechanism makes a reciprocating motion in the vertical direction. Six vibration sensors are arranged vertically on the pump head and the foot of the safe injection pump, and the vibration signal data collected by the sensor are used to evaluate the effectiveness and feasibility of this method in cross-sensor domain migration. Table 10 shows the letters for each measurement point.

The faults used in the experiment are faults that occur naturally during their operation, rather than those that are artificially caused. The failed parts of the failed safe injection pump were used in the experiment and the corresponding data were collected. As shown in Table 10, there are seven types of faults: worm gear poorly engaged, bearing poor lubrication (0 MPa, 17.2 MPa), valve seat compression injury, valve seat erosion, valve seat depression, and gearbox pitting. The operating conditions are the vibration data measured at the measurement point. In the original signal, samples are taken at length 576. To facilitate experimentation, all signals in a certain state are integrated into a column; the label values for each state are from 0 to 7, as shown in Table 11.

#### 4.3.2. Discussion of Batch_Size

A larger batch_size can shorten the training time of each iteration, but it may also reduce the generalization ability, so there should be a balance between the two. Therefore, under the fault dataset with the pump, only the batch_size changes. The results are shown in Table 12.

The training difficulty is not consistent across batches, resulting in different training times. Obviously, it can achieve similar performance (100%, 99.93%) with batch_sizes = 32 or 64, but the latter takes less time, so the batch_size = 64.

#### 4.3.3. Evaluation with Small Samples

**Figure 12:**
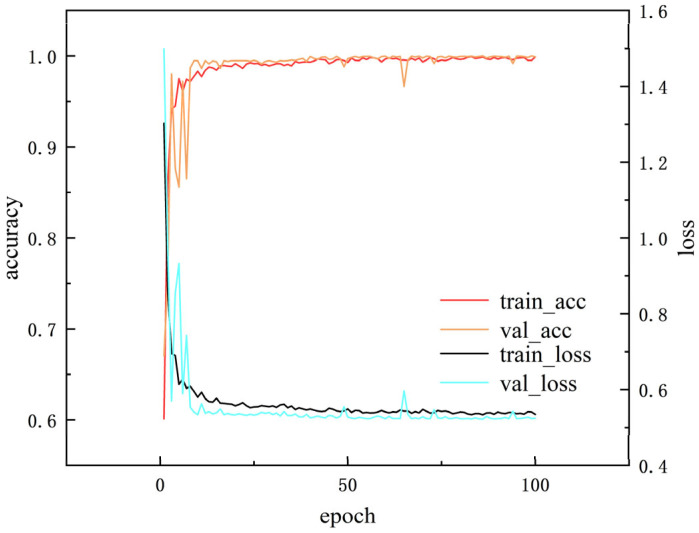
The performance of training and test sets.

Figure 12 reflects the variation curves of accuracy and loss of the training set and validation set when α = 0.4, iteration number =100, and batch_size =64, indicating that AR-CLSTM has good convergence performance. When the iteration number =100, its accuracy can reach 100%.

**Figure 13:**
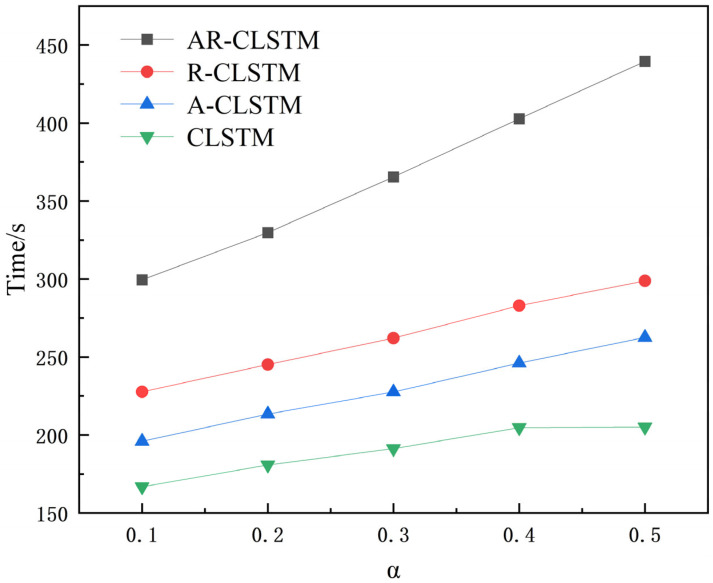
Time of each model under different α.

**Figure 14:**
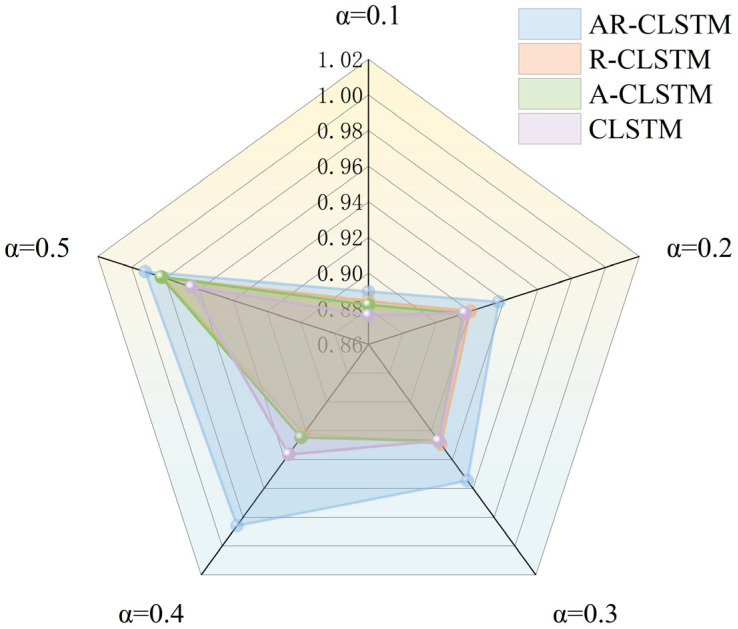
F1 scores with different α.

Figure 13 and Figure 14 show the performance and training time of each model as α increases. It can be seen that the F1 scores of the test set increase as α increases. When α = 0.1, AR-CLSTM has the highest performance, with an F1 score = 0.8897 and CLSTM of 0.8763. At α = 0.5, the performance of all three models except CLSTM is almost 100%. When α < 0.3, CLSTM < A-CLSTM < RCLSTM < CLSTM, and the combination of the attention mechanism and residual block enables the model to achieve optimal performance. However, the cost of this high performance is more training time, so it is required to load the pre-training model to reduce training time.

#### 4.3.4. Visual Analysis

**Figure 15:**
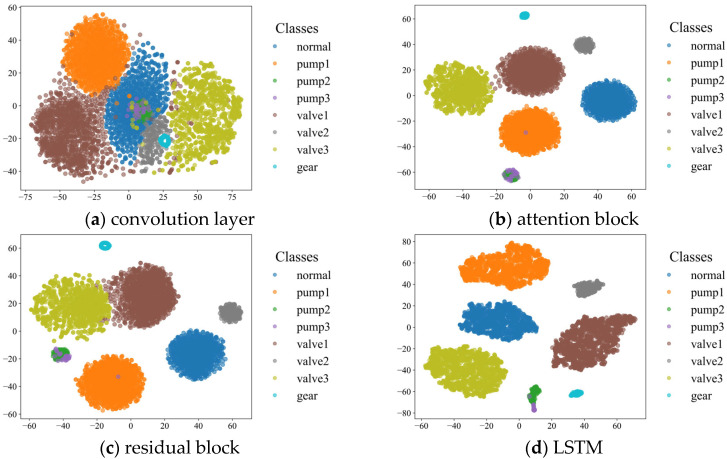
Feature visualization of different layers.

To further reveal the feature representation, we apply the T-SNE technique to feature visualization, where different colors describe different states. By comparing Figure 15a,b, we can find that CNN initially extracts features which are further separated by the attention mechanism of each state. Figure 15b,c show that the attention mechanism and residual block of each state classify the samples by extracting hidden features at different positions. At the same time, it can be found that the classification of small-sample data is more accurate after the model passes through the attention mechanism and residual block. Finally, the information of the whole time series is extracted by LSTM. By comparing LSTM and the attention mechanism, it can be seen that LSTM focuses on the output of neurons in all hidden layers of the model, making the fault state separation more obvious and reducing the training pressure of the diagnostic layer. Meanwhile, from the visualization of each module in Figure 15, it can be seen that AR–CLSTM still has strong robustness in dealing with imbalanced data in the actual industrial environment. The attention mechanism suppresses irrelevant sensor noise and amplifies discriminative features in limited samples. Residual connections enable stable training in shallow layers (one residual block) and avoid overfitting on small datasets. LSR calibrates gearbox pitting (label 7) and bearing lubrication faults (labels 2–3), making it possible to identify the faults. At the same time, from the t–SNE visualization of faults 2, 3, and 7, it can be seen that the proposed model can still accurately identify fault types under conditions with small samples. In summary, AR-CLSTM can better separate different states and has amazing universality.

### 4.4. Comparison of Different Diagnostic Models

Finally, the use of rolling bearing data from CWRU is very popular in mechanical fault diagnosis studies. Compared with some methods listed in Table 13, the optimizers and α values of all algorithms remain consistent, and AR-CLSTM still reached 100% diagnostic performance in the case with no human intervention. Specifically, compared with DRCNN, FasterNet, DRSN, WDCNN, VACNN and RNN-WDCNN, AR-CLSTM is able to achieve an average accuracy gain of 1.86%, 21.86%, 3.57%, 6.14%, 10.71%, and 4%, respectively. Meanwhile, due to the complexity of the model, the computation time exceeds that of the other six algorithms.

**Figure 16:**
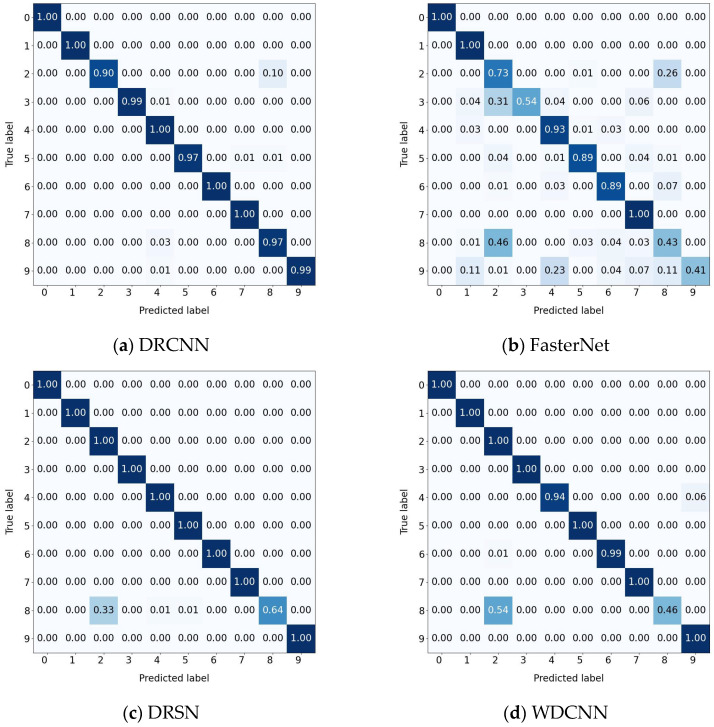

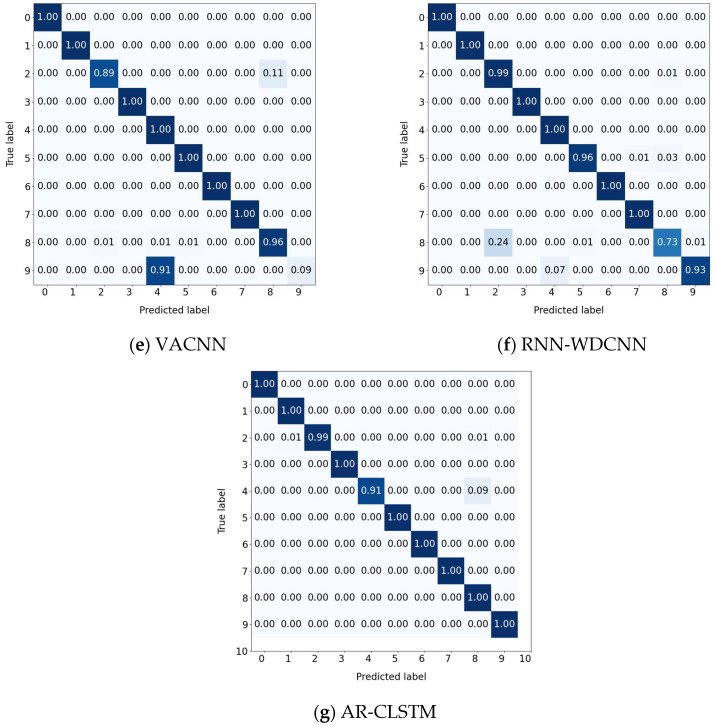
Confusion matrix of different methods: (**a**) DRCNN. (**b**) FasterNet. (**c**) DRSN. (**d**) WDCNN. (**e**) VACNN. (**f**) RNN-WDCNN. (**g**) AR-CLSTM.

The confusion matrix results are depicted in Figure 16. According to the result analysis, other methods clearly tend to misjudge the fault types of labels 2 and 8. This phenomenon may stem from the lack of clear distinguishable features in the original signals of these two states. In contrast, the proposed method effectively enhances the model’s ability to extract discriminative features, thereby improving the overall diagnostic performance.

**Figure 17:**
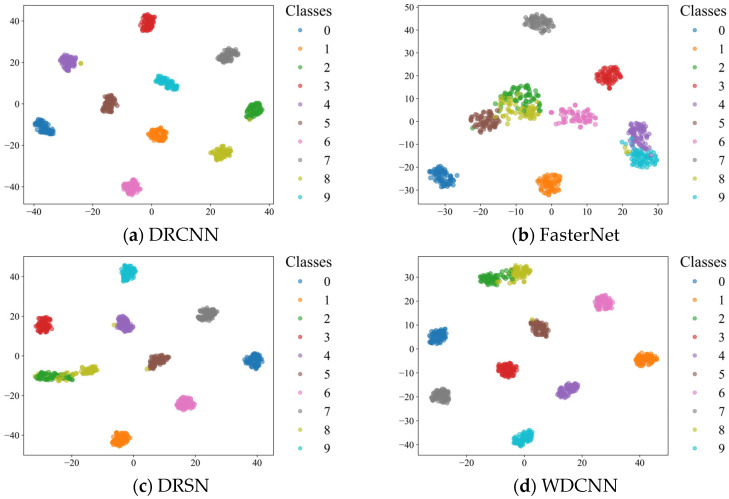

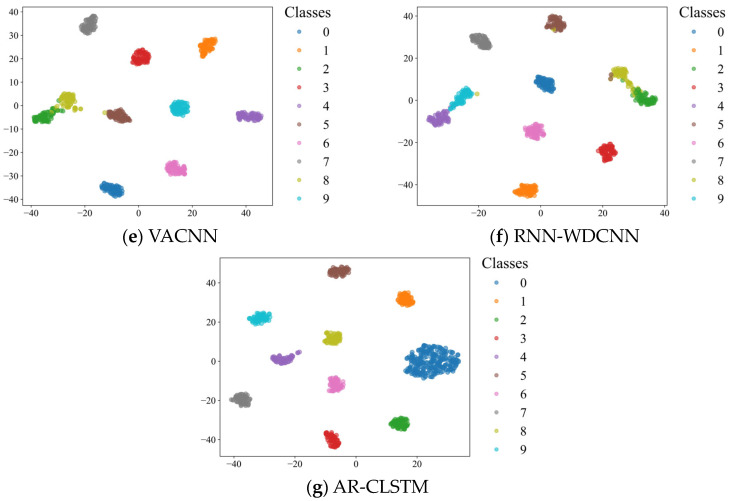
Feature visualization via t-SNE.

To give a more intuitive result, the t-distributed stochastic neighbor embedding (t-SNE) algorithm [39] is introduced to visualize the distributions of the results of the eight methods. As is displayed in Figure 17, each color denotes a health state of the motor. It can be easily found that AR-CLSTM achieves a more discriminative feature distribution map than the other six methods, which further demonstrates the superiority of the proposed method.

## 5. Conclusions

A residual convolutional neural network based on the attention mechanism is put forward for the fault diagnosis of rotating machinery with small samples. The developed attention-reinforced CLSTM architecture demonstrates strong diagnostic capabilities for rotating machinery operating with limited training data. Experimental validation shows this method consistently achieves over 99% accuracy on both CWRU bearing and safety injection pump datasets. This performance advantage comes from combining channel attention mechanisms and spatial attention modules. The channel attention dynamically adjusts frequency-sensitive features, proving particularly effective at identifying subtle fault patterns in pump vibration spectra. Meanwhile, the spatial attention aggregates contextual information across different receptive fields. Together, these components enable reliable feature extraction from small training sets. Our ablation studies confirm that neither attention component alone delivers comparable results. This validates the architectural innovation of integrating both mechanisms. We also examined how the attention mechanism and LSTM layers respond to different training set sizes. To address distribution differences among labeled samples, we implemented label smoothing regularization. Various visualization techniques including t-SNE plots and confusion matrices further demonstrate how AR-CLSTM organizes fault representations hierarchically. Early network layers capture spectral signatures while deeper layers integrate temporal dependencies. Finally, when tested on the CWRU dataset, AR-CLSTM outperformed six other advanced algorithms, showing excellent performance and robustness.

In the upcoming work, we will focus on four key issues: (1) In some industrial scenarios, mechanical signals are often submerged by noise, which makes it difficult to fully utilize the data. We will explore the feasibility of enhancing the diagnostic ability in high-noise scenarios (SNR < 0 dB) by introducing methods such as physical information constraints. (2) Moreover, it is extremely difficult for traditional CNN models to conduct fault diagnosis under the condition of data imbalance. In the future, we will attempt to perform fault diagnosis on mechanical equipment under the imbalanced condition. Due to the advantage of generative adversarial networks (GANs) in generating a small number of samples, we will integrate GANs to synthesize minority-class fault samples to explore the feasibility of fault diagnosis under small-sample conditions after generating minority-class fault samples. (3) Our current architecture optimizes feature extraction within specific spectral regimes but lacks explicit mechanisms for cross-mechanical domain adaptation. To address this, we are developing physics-informed transfer learning modules that decouple machinery agnostic fault patterns from device-specific resonance characteristics. We will include these enhancements in future work to strengthen cross-domain robustness. (4) This study specifically examined model performance under consistent operational conditions using the CWRU dataset as a case study. Our analysis focused on scenarios where training and testing occurred within identical operational environments, represented as A→A and B→B configurations. However, we did not investigate the model’s behavior during cross-operational condition transfer learning. Specifically, performance under scenarios like A→B remains unexplored. To address this limitation in future research, we will implement a domain adaptation module utilizing transformer architecture. (5) We will integrate k-fold cross-validation in subsequent studies, leveraging cloud computing resources. Additionally, we plan to address data imbalance using synthetic minority oversampling or diffusion models.

## Figures and Tables

**Table 1:** Experimental parameter settings.

Setting	Valve
Batch_size	64
Maximum epochs	200
Optimizer	Adam
Learning rate	0.001
Weight decay (except bias)	0.00001

**Table 2:** Model structure and main hyperparameters of the AR-CLSTM model.

No.	Layer Type	Kernel Number	Kernel Size	Activation	Output Shape
1	Input	-	-		(1, 24, 24)
2	Convolution1	64	3 × 3	LeakyReLU	(64, 24, 24)
3	Attention layer1	-	-	-	(64, 24, 24)
4	Residual Block1	-	-	-	(64, 24, 24)
5	Convolution2	64	3 × 3	LeakyReLU	(64, 24, 24)
6	Max-pooling	-	2 × 2	-	(64, 12, 12)
7	Attention layer2	-	-	-	(64, 12, 12)
8	Residual Block2	-	-	-	(64, 12, 12)
9	Convolution3	128	3 × 3	LeakyReLU	(128, 12, 12)
10	Attention layer3	-	-	-	(128, 12, 12)
11	Residual Block3	-	-	-	(128, 12, 12)
…					
12	Convolution6	256	3 × 3	LeakyReLU	(256, 6, 6)
13	Max-pooling	-	2 × 2	-	(256, 3, 3)
14	Attention layer6	-	-	-	(256, 3, 3)
15	LSTM	-	-	Tanh	(256, 10)
16	Fully-connected1	-	-	ReLU	(400)
17	Fully-connected1	-	-	SoftMax	(10)

**Table 3:** Confusion matrix (TN: true negative; FN: false negative; FP: false positive; TP: true positive).

		Prediction Category	
		False	True
Real Category	False	*TN*	*FP*
	True	*FN*	*TP*

**Table 4:** Normalized matrix elements.

		Prediction Category	
		False	True
Real Category	False	*TN*/(*TN* + *FP*)	*FP*/(*TN* + *FP*)
	True	*FN*/(*TP* + *FN*)	*TP*/(*TP* + *FN*)

**Table 5:** Relationship between load and speed.

Type	0 HP	1 HP	2 HP	3 HP
Motor speed	1797 r/min	1772 r/min	1750 r/min	1730 r/min

**Table 6:** Composition of CWRU dataset.

Data	Loads	Locations	FD (mm)	Label	α%
A/B/C/D	0/1/2/3	N	0.007/0.014/0.021	0	0.1~0.5
		IF		1/2/3	
		OR		4/5/6	
		BF		7/8/9	

**Table 7:** Comparison of results for different batch_sizes.

Batch_Size	Training Set	Test Set	Time/s
16	0.9989	0.9993	420.6988
32	0.9925	0.9986	289.3518
64	0.99786	1	130.76886
80	0.99786	0.99929	143.7423
100	0.99679	0.99857	121.6382
128	0.99572	0.99929	117.7134

**Table 8:** Comparison of results under different optimizers.

Optimizers	Accuracy of Training Set	Accuracy of Test Set	Time/s
SGD	0.991	0.93	128.5763
ASGD	0.338	0.292	133.3919
Adamp	0.997	1	252.0788
Adam	0.998	1	130.7688
Rmsprop	0.996	0.999	156.2769
Adamax	0.997	1	153.4686

**Table 9:** Test time under different loads.

Model	α%	Time/s			
		A	B	C	D
CLSTM	0.1	58.60377	68.32886	68.84546	69.01948
	0.2	60.98297	69.53968	70.41173	68.64683
	0.3	62.66856	73.24664	73.26761	73.09081
	0.4	66.42255	78.81137	78.99949	78.61509
	0.5	71.42010	83.74214	82.843	82.51756
R-CLSTM	0.1	73.2638	87.66597	87.47013	90.68301
	0.2	81.50383	93.35573	94.04481	92.89685
	0.3	85.14834	100.3313	100.1096	100.0498
	0.4	92.55912	111.9228	109.0594	110.3115
	0.5	99.55979	115.3333	115.5924	114.9421
A-CLSTM	0.1	63.4881	75.4783	75.7371	75.1743
	0.2	68.9588	80.4958	79.7605	79.1713
	0.3	72.7774	85.9301	85.4315	86.0969
	0.4	78.723	105.1256	93.1588	93.4781
	0.5	84.7834	104.3662	98.8302	98.8531
AR-CLSTM	0.1	96.7927	119.9061	115.5550	113.9553
	0.2	109.1384	125.2133	133.4098	127.1773
	0.3	118.7377	137.1913	138.5698	138.7705
	0.4	129.6907	175.9337	153.0638	153.2145
	0.5	145.0274	180.0862	166.5756	164.0358

**Table 10:** Introduction to measurement points.

Sensor Number	Sensor Location	Sensor Number	Sensor Location
A	Vertical direction of pump head 1	D	Vertical direction of machine foot 1
B	Vertical direction of pump head 2	E	Vertical direction of machine foot 2
C	Vertical direction of pump head 3	F	Vertical direction of machine foot 3

**Table 11:** Safe injection pump database introduction.

Label	Condition	Length	Total Number of Samples
0	Normal	576	1420
1	Worm Gear Poorly Engaged	576	1726
2	Bearing Poor Lubrication(0 MPa)	576	178
3	Bearing Poor Lubrication(17.2 MPa)	576	38
4	Valve Seat Compression Injury	576	1820
5	Valve Seat Erosion	576	310
6	Valve Seat Depression	576	1820
7	Gearbox Pitting	576	92

**Table 12:** Comparison of results between different batch_sizes.

Batch_Size	Training Set	Test Set	Time/s
32	0.999326	0.99865	367.9208
64	0.9993	0.9973	215.4055
80	0.99326	0.994824	185.7243
100	0.993598	0.994824	171.5411
128	0.993261	0.994824	161.5339

**Table 13:** Comparison of fault diagnosis of CWRU.

Models	Optimizer	Loss Function	α	Time/s	Accuracy
DRCNN [33]	Adam	LSR	0.3	22.83077	98.14%
FasterNet [34]	Adam	LSR	0.3	52.34248	78.14%
DRSN [35]	Adam	LSR	0.3	101.9397	96.43%
WDCNN [36]	Adam	LSR	0.3	11.09521	93.86%
VACNN [37]	Adam	LSR	0.3	14.53134	89.29%
RNN-WDCNN [38]	Adam	LSR	0.3	18.14988	96%
AR-CLSTM	Adam	LSR	0.3	130.7688	100%

## Data Availability

Data can be made available upon reasonable request.
